# Incomplete Memories: The Natural Suppression of Tissue-Resident Memory CD8 T Cells in the Lung

**DOI:** 10.3389/fimmu.2018.00017

**Published:** 2018-01-22

**Authors:** Katie L. Reagin, Kimberly D. Klonowski

**Affiliations:** ^1^Department of Cellular Biology, University of Georgia, Athens, GA, United States

**Keywords:** respiratory immunity, influenza infection, CD8^+^ T cells, CD8 memory, heterologous immunity, tissue-resident memory cells

## Abstract

The yearly, cyclic impact of viruses like influenza on human health and the economy is due to the high rates of mutation of traditional antibody targets, which negate any preexisting humoral immunity. However, the seasonality of influenza infections can equally be attributed to an absent or defective memory CD8 T cell response since the epitopes recognized by these cells are derived from essential virus proteins that mutate infrequently. Experiments in mouse models show that protection from heterologous influenza infection is temporally limited and conferred by a population of tissue-resident memory (T_RM_) cells residing in the lung and lung airways. T_RM_ are elicited by a diverse set of pathogens penetrating mucosal barriers and broadly identified by extravascular staining and expression of the activation and adhesion molecules CD69 and CD103. Interestingly, lung T_RM_ fail to express these molecules, which could limit tissue retention, resulting in airway expulsion or death with concomitant loss of heterologous protection. Here, we make the case that respiratory infections uniquely evoke a form of natural immunosuppression whereby specific cytokines and cell–cell interactions negatively impact memory cell programming and differentiation. Respiratory memory is not only short-lived but most of the memory cells in the lung parenchyma may not be *bona fide* T_RM_. Given the quantity of microbes humans inhale over a lifetime, limiting cellular residence could be a mechanism employed by the respiratory tract to preserve organismal vitality. Therefore, successful efforts to improve respiratory immunity must carefully and selectively breach these inherent tissue barriers.

## Introduction

Respiratory infections continue to be one of the leading causes of morbidity and mortality worldwide (1). Approximately four million annual outpatient visits are associated with viral respiratory infections, including influenza and respiratory syncytial virus (RSV) (2, 3). While a RSV vaccine remains elusive, available influenza vaccines induce specific antiviral neutralizing antibodies that recognize the external antigens hemagglutinin and neuraminidase and are protective against a homologous infection. However, host immune pressure promotes mutations of these antigens between seasons rendering the elicited antibodies and those derived from a natural infection ineffective at providing long-term cross-protection against mismatched or heterologous viral strains (3).

Activated CD8 T cells lyse infected lung epithelial cells and produce antiviral cytokines, ultimately eliminating viral reservoirs (4). In the case of influenza infection, CD8 T cells recognize epitopes derived from internal viral proteins that are conserved across 80–100% of circulating influenza strains (4–8), indicating that elicitation of CD8 T cell immunity could offer a broad range of protection against heterologous influenza infection. This protection would rely on the development of memory CD8 T cells (T_mem_) capable of responding rapidly upon challenge (9). However, evidence from murine (6, 10–12) and human (13, 14) studies suggest that long-lived protective T_mem_ does not form in response to influenza infection. While human studies are lacking, murine models indicate that respiratory anti-influenza T_mem_ numbers wane coordinate with loss of heterosubtypic immunity to influenza infection (10). This observation, paired with the knowledge that humans are susceptible to seasonal infections following both natural infection and vaccination with the live, attenuated vaccine (3) shows that respiratory T_mem_ are not stable which we believe is partly due to the incomplete generation of a specific population of T_mem_ in the lung.

## T_RM_: The Other Memory Cell

Infection with various pathogens elicits a heterogeneous T_mem_ pool that was previously thought to consist of predominately two distinct populations: central memory cells (T_CM_) located primarily in lymph nodes and effector memory cells (T_EM_) which circulate through lymphoid and non-lymphoid tissues (15). The preferential localization of T_CM_ is due to expression of CD62L and CCR7 (15), whereas T_EM_ express low levels of these molecules. T_mem_ develop under a transcriptional program regulated by Eomes (16) and require IL-7 signaling for their survival through T cell contraction (17). However, IL-15 and IL-2 signaling bias T_mem_ toward a T_CM_ or T_EM_ lineage, respectively (18). In many cases, T_EM_ provide initial pathogen control at portals of entry, while T_CM_ are positioned to broadly patrol lymph nodes (19). Indeed, T_CM_ provide protection against systemic lymphocytic choriomeningitis virus infection (20), while T_EM_ protect against respiratory Sendai virus challenge (21). However, often this is not a true division of labor and, even in the case of non-lymphoid infections, reactivated T_CM_ will also contribute to the generation of new effector cells, albeit with delayed kinetics.

Subsequent studies using parabiotic mice demonstrated the existence of stationary, non-migratory populations of T_mem_ within the brain and small intestine, and to a lesser extent, other tissues like the lung and liver (22). These cells are now commonly referred to as tissue-resident memory cells (T_RM_). T_RM_ have a core transcriptional profile that distinguishes them from their T_CM_ and T_EM_ counterparts (23), including expression of transcription factor Hobit (24). How T_RM_ cells developmentally diverge from other T_mem_ is unclear; however, it is likely to involve early programming followed by acquisition of tissue-specific factors that promote survival and tissue retention (23, 25). In most cases, CD8^+^ T_RM_ have been identified by expression CD69 and CD103 (αE integrin) which are upregulated on T_RM_ in both humans (26, 27) and mice (28, 29). The ligand of CD103, E-cadherin, is expressed exclusively by epithelial cells and CD69 expression limits tissue egress (30, 31), suggesting these markers are responsible for locking T_RM_ within tissues. In fact, T_RM_ fail to develop in the intestines of CD103^−/−^ mice, and absence of CD69 and CD103 limits T_RM_ formation in the skin (23), indicating that upregulation of CD103 and CD69 are crucial steps for the establishment of T_RM_. Expression of CD103 and CD69 is regulated by TGF-β (32), which is highly expressed in mucosal sites such as the gut (33) where stable populations of T_RM_ cells have been observed (34). In most cases, T_RM_ are maintained through IL-7- and IL-15-mediated homeostatic proliferation (35, 36). T_RM_ are confirmed to exist in the skin (28, 37), brain (38), liver (39), and female reproductive tract (40, 41) where they are stably maintained. T_RM_ can persist for up to 120 days in the brain following vesicular stomatitis virus (VSV) infection (38), and skin-resident T_RM_ are the most durable, up to a lifetime in mice following cutaneous herpes simplex virus infection (42).

While a secondary, recall response can be delayed by several days for the activation of T_mem_ and recruitment of new effectors to the infection site, T_RM_ respond immediately to pathogen re-exposure (12). Upon antigen re-encounter, T_RM_ produce IFN-γ (9) to recruit circulating T_EM_ and other immune cells from the blood (43). In addition, T_RM_ can directly kill target cells *ex vivo* (44), suggesting a cytotoxic potential. T_RM_ have been shown to mediate long-term protection *in vivo* to infections in the intestine (34), female reproductive tract (40, 41), brain (45), and skin (28, 37). Regarding the latter, the smallpox vaccine, administered by skin scarification, generated T_mem_ which survived for decades (46). While the specific role of T_RM_ in the success of this vaccine is unclear, mice vaccinated *via* scarification of recombinant vaccinia virus (VacV) generate skin-resident T_RM_ that mediate protection against subsequent VacV infection (47). However, not every infection generates stable T_mem_ pools. While T_RM_ cells populate the lung and lung airways after influenza infection (12), protection between influenza seasons following natural infection or vaccination with the live-attenuated vaccine is lost (3), suggesting T_RM_ responses may be uniquely regulated in the lung.

## T_RM_ in the Lung

T_RM_ cells exist within the lung in two distinct compartments: the lung airways and the lung parenchyma. Influenza-specific airway-resident T_RM_ are CD11a^lo^CXCR3^hi^ (48, 49) and can be isolated by bronchoalveolar lavage. It is estimated that anti-influenza T_RM_ in the lung airways have a half-life of only 14 days, and for some period of time are continually replenished from the circulating T_EM_ pool (48). Interestingly, airway T_RM_ have a low cytolytic capacity and fail to proliferate upon antigen re-encounter but rapidly produce antiviral cytokines such as IFN-γ (44). T_RM_ embedded in the lung parenchyma are CD11a^hi^CXCR3^lo^, highly cytolytic and undergo rapid proliferation after antigen re-exposure (44). We have known for some time that regional T_mem_ are responsible for limited heterologous immunity after respiratory infection (10). A careful study of the kinetics of T_mem_ decay after Sendai and influenza virus infections demonstrated a rapid decline in T_mem_ numbers in the lung and lung airways by 90 days postinfection. Importantly, this loss of influenza-specific T_mem_ in the lung coincided with loss of heterosubtypic immunity to influenza infection (10). The attrition of influenza-specific cells is restricted to the lung, as splenic memory cell numbers do not decline, indicating this is likely loss of the T_EM_ or T_RM_ pools. Subsequent experiments demonstrated that airway CD103^+^ cells are responsible for protection against a secondary, heterologous virus challenge. However, this pool declines rapidly after infection and is undetectable within 7 months postinfection (12), in part due to the inhospitable environment of the lung airways.

T_RM_ in the airways reside at the frontline, adjacent to influenza-susceptible epithelial cells. However, lung parenchymal T_RM_ and circulating T_EM_ are also available within the lung tissue and can serve as a secondary line of defense. Recent evidence indicates that over time, T_RM_ cells in the lung airways wane and are replaced by circulating T_EM_ cells; however, these T_EM_ also decline and lose the ability to convert to T_RM_ (50). This, coupled with a loss of T_RM_ in the lung parenchyma, results in a gradual decline in the overall T_RM_ population in the lung. Decline in the lung parenchymal T_RM_ pool could be due to increased cell death, limited proliferation, or emigration. Unlike T_RM_ in other sites (28, 34, 38), most lung T_mem_ do not undergo homeostatic proliferation (50, 51). However, a small pool is replenished from proliferating T_mem_ that have recently emigrated from secondary lymphoid tissues (50). In addition, there is no evidence that T_RM_ cells in the airways egress from the lung or re-enter circulation (48). Therefore, we propose that T_mem_ embedded in the lung tissue are either eventually lost to the airways or do not represent a *bona fide*, protective T_RM_ pool. Our opinion that lung parenchymal T_RM_ do not exist is based on two observations. The first is that few T_mem_ truly penetrate into the tissue and the second is that those T_mem_ that do, are not CD103^+^CD69^+^.

**Figure 1:**
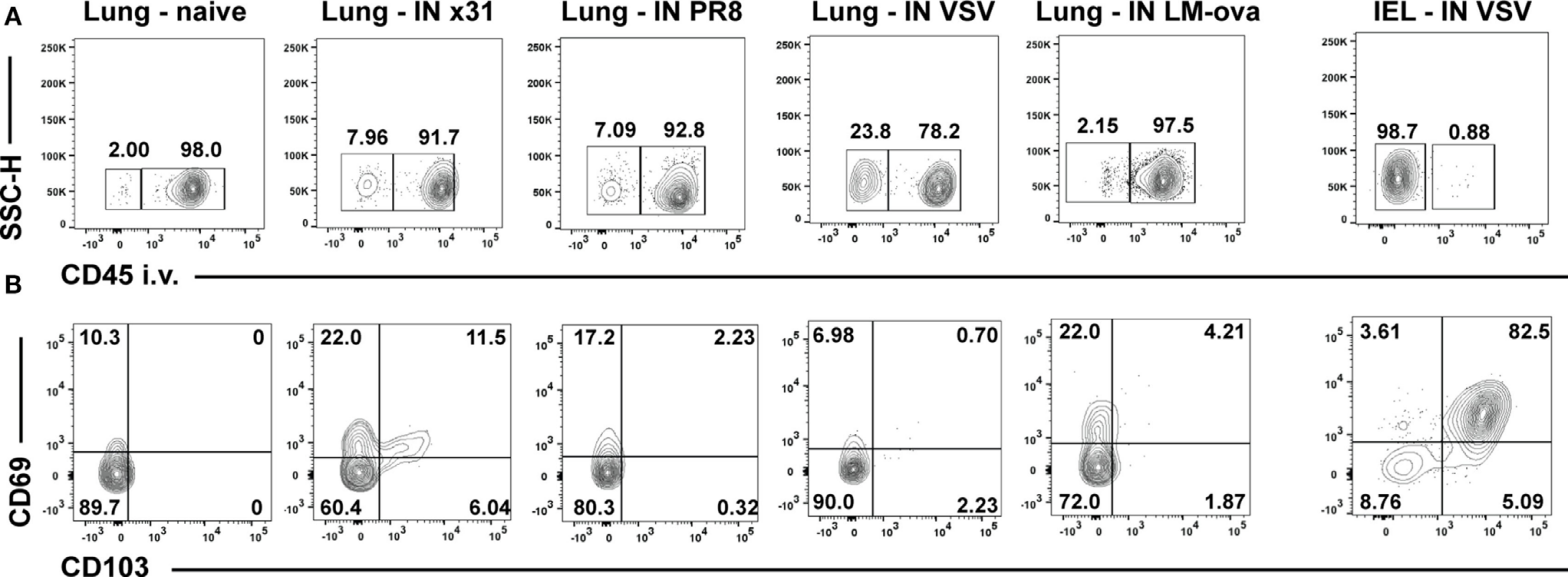
Lung T_RM_ cells express low levels of CD69 and CD103 after respiratory infection with various pathogens. Age- and sex-matched C57/BL6 mice were infected intranasally with a 50-μl inoculum of PBS alone (naïve) or containing sublethal doses of either influenza (10^3^ pfu of strain HKx31 and 10 pfu of PR8), VSV (10^4^ pfu, Indiana strain), or *Listeria monocytogenes* expressing the recombinant ovalbumin (ova) (LM-ova) (10^4^ cfu). One group of mice was additionally intravenously (i.v.) infected with 10^4^ pfu VSV. Animals were sacrificed 35 days later and T_RM_ assessed by intravascular staining. Briefly, mice were injected i.v. with 3 μg FITC labeled αCD45 antibody 3 min before sacrifice, lungs or small intestine were harvested, and lymphocytes isolated as previously described (22). **(A)** Representative i.v. staining of lymphocytes isolated from the lungs or intraepithelial lymphocytes (IEL) of naïve mice or following the indicated infections. All samples were first gated on CD8^+^CD44^+^ memory phenotype cells and gates in **(A)** were set by FMO controls within each experiment. For the influenza and VSV-infected animals, an additional MHC-class I tetramer gate was applied to identify antigen-specific CD8 T cells [as in Ref. (61)]. Numbers in the right box represent the frequency of the gated cells that stained with the i.v. injected antibody (αCD45-FITC^+^) and are in the vasculature (IV^+^). **(B)** Representative CD103 and CD69 staining of IV^−^ [resident cells, left box in **(A)**] cells from the various infections.

Many techniques can identify T_RM_ (Table 1) and each has pros and cons. We believe that the most effective methodology is the combination of two of these approaches: intravascular staining and CD103/69 phenotyping. Intravascular staining distinguishes between cells circulating through the blood and those embedded within a tissue (52). Approximately 99% of the T_RM_ within the epithelial layer of the small intestine are protected from the intravascular staining (Figure 1) (52, 53), validating similar results observed in parabiotic mice (22). In contrast, the majority of the memory cells within the lung parenchyma 35 days after respiratory infection with either influenza, VSV, or *Listeria monocytogenes* are part of the circulating T_EM_ pool, with only 10–20% of the cells in the lung parenchyma truly within the tissue (52) (Figure 1). These data do contrast with other respiratory infections that are skewed toward the upper respiratory tract (54) or are chronic (55), both cases generating CD103^+^CD69^+^ T_RM_. With regard to the latter study, it is possible that persistent antigen and inflammation is required for the successful development of T_RM_ within this site. In addition to antigen access, antigen competition can regulate T_RM_ populations at the clonal level (56). Moreover, many studies identify lung T_RM_ *via* CD103 and CD69 expression on isolated lymphocytes (57, 58), independent of intravascular staining. However, expression of these markers does not always correlate with tissue residency. For example, some T_RM_ cells in the lamina propria of the gut (59), the liver (39), and the brain (60) are CD103^−^, and human splenic T_mem_ can be CD69^+^ (26). In fact, less than 30% of the IV protected T_RM_ cells isolated from the lung parenchyma express CD69 and CD103 (Figure 1) compared to T_RM_ isolated from other mucosal sites, where expression ranges from approximately 50–99% (59). Therefore, T_mem_ located in the lung parenchyma after respiratory infection lack one of the key attributes associated with *bona fide* T_RM_, expression of CD69 and CD103. CD103^−^ T_RM_ in the brain are maintained for a few months (60) which may be due to modified tissue localization and enhanced access to IL-15. However, lung parenchymal T_RM_ are maintained independent of IL-15 (61), at least in the short-term, so gained proximity to IL-15 may not matter. However, acquisition of other survival signals dependent on CD103 positioning may be altered, leading to either cell death or assimilation into the T_EM_ pool. Coupled with loss of airway-associated T_RM_, this situation leaves circulating T_EM_ as the only viable responders. Whether the T_EM_ temporally supplementing the T_RM_ pool are CX_3_CR1^hi^ and classified as the recently described “peripheral” memory cells (T_pM_) (62, 63) is unknown. Nonetheless, as T_EM_ induced from respiratory infection decline over time (64), hosts will then be susceptible to infection. Therefore, an inferior CD69^+^CD103^+^ T_RM_ response underpins loss of heterosubtypic immunity in the lung and raises the question of why long-lived, stable T_RM_ does not form in the lung following respiratory infection.

**Table 1:** Common methods used for the identification of T_RM_ cells in peripheral sites.

Technique	Strengths	Weaknesses
Intravascular staining (Intravascular staining followed by flow cytometry)	Identifies cells circulating within the bloodstream, eliminating contamination of parenchymal T_RM_ by T_EM_ within the intervening vessels, and eliminating the need for tissue perfusion (65) Methodology highlights cellular location, which defines T_RM_ (52, 54, 58)	Labor intensive (requires careful timing of Ab injection and animal sacrifice) (65) Extensive tissue digestion protocols can result in inefficient cell isolation that can skew T_RM_ representation Differential kinetics of antibody vascular extravasation or blood flow rates within specific tissue can affect antibody penetrance (66, 67) Identifies localization at a single point in time; cannot eliminate transient migration through tissue
CD69/CD103	Simple method of detection by flow cytometry on isolated tissue lymphocytes *ex vivo* (29)	Extensive tissue digestion protocols (see above) Not exclusively expressed on cells in tissue parenchyma (59) CD69 expression is enriched in conditions of antigen persistence (68) Requires perfusion to eliminate tissue-associated cells in vasculature (69) Cells are not uniformly CD69/CD103+ in all tissues (59)
Confocal microscopy	Clearly identifies cells directly embedded in parenchyma or epithelium while excluding those in small vessels (57, 58) Can reveal T_RM_ tissue niche (58) Can identify which cells T_RM_ are interacting with (59)	Cryosectioning can damage or distort tissue architecture (70) Information is only a snapshot and limited tissue depth (70)
Parabiosis	Identifies the proportion of circulating T_mem_ in a given tissue (using congenic markers of partner) in the steady state (22)	Requires surgical procedure and extensive animal monitoring (71) Unclear how much inflammation due to surgery changes T_mem_ cell migration/redistribution of subtypes (71) Cannot distinguish between host T_RM_ and T_EM_ without pairing with other technique (22, 72)
FTY720 treatment	Eliminates the ability of circulating T_mem_ to traffic into tissues and supplement the T_RM_ pool (enriches for T_RM_) (12, 28)	Does not eliminate the contribution of circulating memory cells (T_EM_) in the blood before lymph node sequestration (73)

*A summary of some of the commonly used immunological techniques that have been used to study T_RM_ cells in various peripheral sites, as well as the strengths and weaknesses of said techniques. With the exception of confocal microscopy, these techniques do not consider lung compartmentalization, which requires additional processing of BAL and subsequently lung tissue to identify the different T_RM_ pools*.

## The Respiratory Environment Subverts the Development of T_RM_

As the lung is exposed to both infectious agents and innocuous environmental antigens, immune responses must be tightly controlled to prevent immunopathology (25). Similar regulation is also required in the liver and brain, additional tolerogenic sites. In part, this regulation is accomplished *via* tissue segregation. Indeed, liver T_RM_ are exclusively segregated from tissue stroma, retained within the sinusoids (74), whereas brain T_RM_ are preferentially localized in the meninges and perivascular areas (60), sequestered from the parenchyma. The lung is no different, with the development of BAL T_RM_ and parenchymal T_RM_. However, unlike T_RM_ in the brain and liver, BAL T_RM_ are directly exposed to the external environment and easily lost, whereas the lung parenchymal T_RM_ are imbedded in the parenchyma and require an additional level of regulation to prevent immunopathology.

One potential mechanism is through altered mammalian target of rapamycin (mTOR) signaling within the respiratory tract. mTOR is responsible for regulating cellular metabolism, proliferation, and differentiation (75), including memory cell development (76). High levels of mTOR activation reduces the total number of antigen-specific cells expressing CD127, required for the development of memory precursor cells (77), and the subsequent T_CM_ pool (76). While reducing mTOR signaling with rapamycin reverses the effects on T_CM_ (76), T_RM_ formation and retention within the intestinal mucosa was also increased *via* enhanced expression of gut-specific homing molecules (78). To date, no study has linked reduced mTOR signaling to enhance lung homing and/or *respiratory* T_RM_ formation. However, evidence from viral respiratory infection models support a role for mTOR in T_RM_ formation. Rapamycin treatment during influenza infection increases the total number of antigen-specific CD8 T_mem_ circulating in the blood (79) similar to studies in the gut (78). In addition, activated CD8 T cells isolated from infants infected with RSV and treated with rapamycin during *in vitro* re-stimulation express higher levels of CD127 compared to those cells stimulated without rapamycin. Rapamycin treatment also enhanced the effector response of RSV-specific cells by increasing their proliferation and production of granzyme B (80). While increased infiltration of RSV-specific effector cells into the lung may be important for viral clearance, this can also result in damaging pathology within the lung tissue itself. This indicates that perhaps careful regulation of mTOR signaling during respiratory infection is important for limiting potential immunopathology (80) and T_mem_ development; however, further studies are needed to directly implicate mTOR as a player in lung T_RM_ formation.

The lung environment is inherently immunosuppressive. In the steady state, a large reservoir of T_regs_ populate this tissue and contribute to significant IL-10 post-influenza infection (81). Moreover, bronchial and alveolar epithelial cells are known to express moderate levels of the programmed death-1 (PD-1) ligands PD-L1 and PD-L2, both of which are significantly upregulated upon RSV (82) and influenza infection (83). In addition, antigen-specific CD8 T cells infiltrating the lung following RSV and influenza infection have an increased expression of PD-1 (83, 84). Both IL-10 and PD-1 signaling can modulate CD8 T cell activation both individually (85, 86) and cooperatively (87) by tuning TCR signaling. IL-10 suppresses IL-12 signaling which, like PD-1 signaling, activates mTOR. However, PD-1 signaling is not exclusively through mTOR and can affect transcriptional networks and other cell cycle regulators which can impact the fate and function of CD8^+^ T cells (86). Memory phenotype cells isolated from PD-1^−/−^ versus wild-type mice are preferentially T_EM_ (88). Reciprocal adoptive transfer experiments demonstrated this bias was inherent to the T cell. As PD-1 blockade during RSV infection results in enhanced inflammation and lung injury, PD-1/PD-L1 expression in the respiratory tract may serve to limit the expanding CD8^+^ T cell pool, thereby restricting developing T_RM_. Thus, while enhanced PD-1 expression within the respiratory tract may be important for regulating inflammation, this may create an environment that is inhospitable to the formation of T_RM_.

It is also possible that respiratory infections alter T_RM_ programming *via* inhibition of CD103 and CD69 expression, which negatively affects the formation and/or retention of T_RM_ cells in the respiratory tract. Constitutive expression of TGF-β in mucosal sites such as the gut (33) is crucial for the development of long-lived T_RM_ through induction of CD103 expression (89). Epithelial cells also provide survival signals such as IL-15 (90), thus high CD103 expression may not only facilitate T_RM_ retention but aid in their development or survival *via* tissue positioning. However, high levels of TGF-β in the respiratory tract can be detrimental, leading to the development of cystic fibrosis within the lung (91). Although TGF-β expression is induced by influenza infection (92, 93), it may only be transiently expressed to limit immunopathology, albeit at the expense of T_RM_ formation. In fact, the T_RM_ in peripheral sites can cause semi-permanent scarring in tissues that worsens after T_RM_ re-activation and production of IFN-γ *in situ* (94). Since high levels of IFN-γ production (95), in addition to scarring and fibrosis in the lung, can cause respiratory failure (96), the retention of T_RM_ long term may be inherently limited to maintain host fitness. If this is the case, promoting T_RM_ formation within the respiratory tract could have severe consequences for host respiratory health. Therefore, by reducing TGF-β, and coordinately CD103 expression, lung memory precursor cells would perhaps be ill positioned to receive homeostatic signals responsible for the development, survival, and/or retention of T_RM_ and could be either be lost or assimilated into the T_EM_ pool.

While airway-resident T_RM_ cells confer protection against secondary influenza infection, they rapidly wane, leaving only parenchyma resident T_RM_ and circulating T_EM_ to maintain protection against subsequent infection. However, T_EM_ also wane over time (64) and the formation of *bona fide* T_RM_ in the lung parenchyma is limited (Figure 1). These incomplete memories leave the host susceptible to recurring influenza infection. We believe the lung evokes a form of natural immunosuppression whereby inhibitory signals in the site protect the host from debilitating tissue damage while simultaneously suppressing the formation of *bona fide* T_RM_ within the lung tissue. While the exact mechanisms that underlie altered T_RM_ formation within the respiratory tract are still not fully understood, future efforts to improve the maintenance and stability of this population must bear caution due to potentially negative, long-term effects on the host. Moreover, in developing vaccines against respiratory pathogens, it will be important to identify strategies that will prevent re-infection with respiratory viruses without compromising host respiratory health.

## Ethics Statement

All animal studies were conducted under guidelines approved by the Institutional Animal Care and Use Committee of the University of Georgia.

## Author Contributions

Both KK and KR conceived and wrote the perspective.

## Conflict of Interest Statement

The authors declare that the research was conducted in the absence of any commercial or financial relationships that could be construed as a potential conflict of interest.
